# Washed Microbiota Transplantation Improves Patients with Overweight by the Gut Microbiota and Sphingolipid Metabolism

**DOI:** 10.3390/biomedicines11092415

**Published:** 2023-08-29

**Authors:** Lei Wu, Zi-Han Lin, Xin-Jian Lu, Xuan Hu, Hao-Jie Zhong, De-Jiang Lin, Tao Liu, Jia-Ting Xu, Wen-Ying Lin, Qing-Ping Wu, Xing-Xiang He

**Affiliations:** 1 Department of Gastroenterology, Research Center for Engineering Techniques of Microbiota-Targeted Therapies of Guangdong Province, The First Affiliated Hospital of Guangdong Pharmaceutical University, Guangzhou 510080, China; wuleigdim@163.com (L.W.); z15917376129@163.com (Z.-H.L.); 13420179256@163.com (X.-J.L.); xuan_hu0625@126.com (X.H.); jaxzhong@126.com (H.-J.Z.); 18702040027@163.com (W.-Y.L.); 2 Guangdong Provincial Key Laboratory of Microbial Safety and Health, State Key Laboratory of Applied Microbiology Southern China, Institute of Microbiology, Guangdong Academy of Sciences, Guangzhou 510070, China; 3 School of Biology and Biological Engineering, South China University of Technology, Guangzhou 510006, China

**Keywords:** fecal microbiota transplantation (FMT), washed microbiota transplantation (WMT), overweight and obesity, sphingolipid metabolism

## Abstract

Background: Overweight (OW) and obesity have become increasingly serious public health problems worldwide. The clinical impact of washed microbiota transplantation (WMT) from healthy donors in OW patients is unclear. This study aimed to investigate the effect of WMT in OW patients. Methods: The changes in body mass index (BMI = weight (kg)/height (m)^2^), blood glucose, blood lipids and other indicators before and after WMT were compared. At the same time, 16S rRNA gene amplicon sequencing was performed on fecal samples of OW patients before and after transplantation. Finally, serum samples were tested for sphingolipids targeted by lipid metabolomics. Results: A total of 166 patients were included, including 52 in the OW group and 114 in the normal weight (NOW) group. For OW patients, WMT significantly improved the comprehensive efficacy of OW. In the short term (about 1 month) and medium term (about 2 months), a significant reduction in BMI was seen. At the same time, in the short term (about 1 month), liver fat attenuation (LFA), triglyceride (TG) and fasting blood glucose (FBG) were significantly reduced. In the long term (about 5 months), total cholesterol (TC), low-density lipoprotein cholesterol (LDL-c), non-high-density lipoprotein (non-HDL-c), etc. were significantly reduced. WMT improved the gut microbiota of OW patients, and also had an improvement effect on OW patients by regulating sphingolipid metabolism. Conclusion: WMT had a significant improvement effect on OW patients. WMT could restore gut microbiota homeostasis and improve OW patients by regulating sphingolipid metabolism.

## 1. Introduction

Overweight (OW) and obesity are considered an epidemic of the 21st century, contributing to type 2 diabetes (T2DM), metabolic-associated fatty liver disease (MAFLD), cardiovascular disease (CAD), etc. These obesity-related metabolic diseases can reduce life expectancy [1]. It was estimated that from 1990 to 2015, there was a relative 28.3% increase in mortality associated with high body mass index (BMI) worldwide, with nearly 70% of high-BMI-related deaths due to cardiovascular disease [2]. The complex etiology and pathogenesis of obesity and its related metabolic abnormalities require consideration of multiple factors such as socioeconomic, environmental, individual behavior and heredity factors, which pose great challenges to diagnosis and treatment.

Bioactive sphingolipid (SPL) appears to act as a novel biomarker for overweight and obesity [3]. A large number of studies have shown that SPL can regulate inflammatory response [4]. It has been suggested that ceramides are some of the main categories of the sphingolipid family and may play a pathogenic role [5]. One study suggested that dihydroceramide and hemophosphatediol could serve as novel biomarkers to identify people at high risk for diabetes [6]. Certain types of ceramides are associated with various cardiometabolic diseases [7,8]. These observations add to the interest in and diagnostic potential of sphingolipids in the pathophysiology of disease.

The gut microbiota of obese patients has characteristic changes in composition and function. It was found that the diversity of gut microbiota in obese patients decreased [9]. Most studies have suggested that low bacterial richness is also associated with obesity-related metabolic abnormalities [10]. With further research, specific changes in gut microbiota are expected to be used to diagnose and predict obesity-related metabolic diseases. Fecal microbiota transplantation (FMT) is a novel technique that uses the healthy gut microbiota of a healthy person to replace the disturbed gut microbiota of a patient [11]. FMT is now considered as a standard treatment guideline for cases of recurrent *Clostridium difficile* infection [12]. FMT is gaining increasing interest among researchers [13]. FMT is currently used in a wide range of diseases, such as inflammatory bowel disease [14], Crohn’s disease [15] and functional gastrointestinal disorders [16], which are also associated with significant ecological disorders. Whether FMT can improve overweight remains to be discussed in clinical medicine. Washed microbiota transplantation (WMT) is similar to traditional FMT in principle, but the difference between WMT and traditional FMT is that a fecal bacteria intelligent separation system is used to obtain fecal bacteria and repeated washing reduces harmful substances in fecal bacteria. WMT has been added as a safety measure. It has good safety, quality control and effectiveness against diseases with floral disorders [17].

We attempted to observe whether WMT has an improvement effect on overweight patients. Therefore, we conducted a retrospective study. We collected data according to the actual situation of the hospital system and then performed statistical analysis in the original situation to show the most realistic data.

## 2. Materials and Methods

### 2.1. Patients and Experimental Design

This study included patients who received WMT treatment for functional bowel disease, gastroesophageal reflux disease (GERD) or functional dyspepsia and other diseases in our hospital from December 2016 to May 2022 and completed 1–3 courses of treatment. Inclusion criteria: over 18 years of age, willing to accept WMT. Exclusion criteria: less than 18 years old before transplantation, pregnant women, BMI < 18.5 kg/m^2^ and the use of hypoglycemic, lipid-lowering and blood pressure drugs, weight-loss drugs and related bariatric surgery during the course of treatment. Finally, a total of 166 patients met the requirements.

Among the diagnostic criteria for the overweight group (OW group), BMI ≥ 24.0 kg/m^2^ was defined as overweight. For the normal body weight group (NOW group): 18.5 ≤ BMI ≤ 23.9 kg/m^2^ [18,19]. In the end, 52 people were included in the overweight group and 114 in the normal-weight group.

### 2.2. Preparation of Washed Microbiota and WMT Process

The WMT procedure was in line with the Nanjing Consensus on Washed Microbiome Transplantation Methodology [20]. The washed bacterial solution was prepared by an intelligent microbial separation system (GenFMTer). The center implemented the standard of “three and three courses of treatment” of WMT. The transendoscopic enteral tubing (TET) was placed in the lower digestive tract through colonoscopy. The study was divided into the baseline period, short term (about 1 month from the baseline), medium term (about 2 months from the baseline) and long term (about 5 months from the baseline). All patients received at least 2 WMTs (at least short-term completion of WMT) and completed follow-up.

### 2.3. Clinical Data Collection

Medical records were collected for baseline values and short-, medium- and long-term outcomes before treatment. Data included age (years), sex *n* (%), smoking history, alcohol consumption history, BMI (kg/m^2^), disease or indication of WMT and laboratory test results. They mainly included liver function: liver fat attenuation (LFA, dB/m), liver stiffness measurement (LSM, kPa; blood lipid index: total cholesterol (TC, mmol/L), triglyceride (TG, mmol/L), low-density lipoprotein cholesterol (LDL-c, mmol/L), high-density lipoprotein cholesterol (HDL-c, mmol/L), apolipoprotein B (ApoB, g/L), non-high-density lipoprotein (non-HDL-c, mmol/L), lipoprotein a (LIP, mmol/L); blood glucose index: fasting blood glucose (FBG, mmol/L), HbA1c (%); insulin index: fasting insulin (FI, μU/mL), insulin resistance value (HOMA-IR, insulin resistance value = fasting blood glucose × fasting insulin/22.5); blood pressure indicators: systolic blood pressure (SBP, mmHg) and diastolic blood pressure (DBP, mmHg) upon admission. Adverse events (AEs) included: abdominal pain, diarrhea, nausea and vomiting, dizziness, fatigue, etc. After all patients received WMT treatment and completed follow-up, the results of BMI, blood lipid, blood glucose, blood pressure and other results were statistically analyzed and evaluated.

### 2.4. DNA Extraction and Sequencing

Stool samples were collected from 5 overweight patients, 5 normal-weight patients and 5 donors before and after WMT for sequencing. All samples were stored at −80 °C after collection until DNA extraction. Microbial DNA was extracted using the QIAamp DNA stool mini kit (QIAGEN, Hilden, Germany) [21]. DNA quality and concentration were examined by a NanoDrop™ 2000 (Thermo Fisher Scientific, Wilmington, DE, USA). Primers 338F (5′-ACTCCTACGGGAGGCAGCAG-3′) and 806R (5′-GGACTACHVGGGTWTCTAAT-3′) were used for PCR amplification of bacterial 16S rRNA gene fragments (V3–V4) from extracted DNA. The PCR products were subjected to agarose gel electrophoresis to determine the amplicon size. The constructed library was quantified by Qubit. After the library was qualified, a NovaSeq6000 (Illumina, San Diego, CA, USA) sequencing platform was used for machine sequencing.

### 2.5. Amplicon Data Processing and Analysis

From all the sample data split from plane data and amputation of barcode and primer sequences after the use of FLASH software (version 1.2.11, http://ccb.jhu.edu/software/FLASH/) to splice the sample reads, raw tags were obtained [22]. Then, fastp software version 0.23.1 (Shenzhen Hypros, Shenzen, China) was used to obtain high-quality clean tags [23]. Finally, clean tags were compared with the database to detect and remove chimeras, so as to obtain the effective tags [24]. The DADA2 Variants in QIIME2 were used to obtain the final ASV variants and feature lists for the variants. The resulting ASVs were then compared with the database using the classify-sklearn module in QIIME2 software version 2.0 (QIIME 2 development team, https://docs.qiime2.org) to obtain species information for each ASV.

### 2.6. Extraction and Data Analysis of Lipidomics and Sphingolipomics

Serum samples from 9 overweight patients and 9 normal-weight patients were collected before and after WMT for lipidomics studies based on liquid–mass combination (LC-MS) techniques. Metabolite extraction: a 100 μL liquid sample was added to a glass centrifuge tube with a Teflon-lined cap and 0.75 mL of precooled methanol was added and vortexed. Then, 2.5 mL of precooled methyl tert-butyl ether was added and incubated at room temperature in a shaker for 1 h. Then, 0.625 mL mass spectrum grade water was added and mixed well and the organic phase was stratified, incubated at room temperature for 10 min and centrifuged 1000× *g* for 10 min. The upper organic phase (MTBE) was collected, and 1 mL of mixed solvent (methyl tert-butyl ether/methanol/water (10:3:2.5, *v*/*v*/*v*) was added to the lower layer (water and methanol). The organic phases that were collected twice were enriched by a nitrogen-blowing apparatus [25]. Resolution was performed with 100 μL isopropyl alcohol and then analyzed by an LC-MS/MS system. Data analysis: raw data files were imported into Compound Discoverer (CD) repository search software version 3.1 (Thermo Fisher Scientific, Bohemia, NY, USA). The retention time, mass/charge ratio and other parameters were simply screened. Then, different samples were aligned, peaks were extracted and, at the same time, peak area was quantified, then the target ion was integrated. The molecular formula was predicted by molecular ion peaks and fragment ions and compared with Lipidmaps and Lipidblast databases. Blank samples were used to remove background ions. The quantitative results were normalized and the lipid data results were qualitatively and quantitatively analyzed.

### 2.7. Data Analysis

Statistical analysis was performed using SPSS 22.0 (IBM Corp., Armonk, NY, USA) and Prism 8 (GraphPad, San Diego, CA, USA). The results are expressed as frequency and percentage of categorical variables and mean and standard deviation of continuous variables with normal distribution. Categorical variables were analyzed using chi-square or Fisher exact tests. In univariate analysis, the statistical significance (*p* value) of metabolites between the two groups was calculated based on the *t*-test and the fold change (FC value) of metabolites between the two groups was calculated. The default criterion for differential metabolite screening was VIP > 1, *p* value. The volcano map was drawn with the R package ggplot2, and the VIP value, log2 (fold change) and −log10 (*p* value) of metabolites could be integrated to screen the metabolites of interest. A two-tailed *p* value < 0.05 was considered statistically significant.

## 3. Results

### 3.1. Clinical Features of Patients Receiving WMT

**Figure 1:**
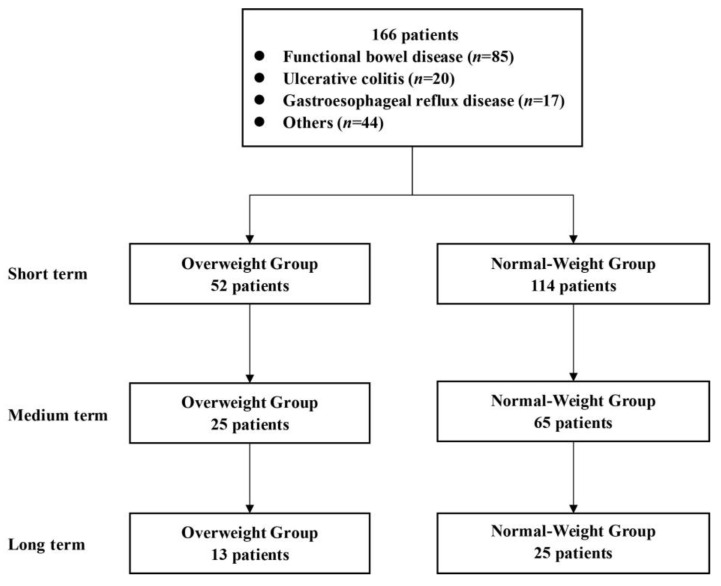
Flow chart of this study.

From December 2016 to May 2022, WMT was completed in the First Affiliated Hospital of Guangdong Pharmaceutical University. There was a total of 166 patients (52 patients in the overweight group and 114 patients in the normal-weight group) who met the enrollment criteria, including 83 males (50%) and 83 females (50%). The mean ± standard deviation of age was 51.92 ± 15.84 years old. The analysis process is shown in Figure 1. Table 1 shows the top six disease characteristics of patients undergoing WMT, which were functional bowel disease (*n* = 85, 51.20%, including irritable bowel syndrome, functional constipation and functional diarrhea), ulcerative colitis (*n* = 20, 12.05%), gastroesophageal reflux disease (*n* = 17, 10.24%), non-alcoholic fatty liver disease (*n* = 8, 4.82%), atopic dermatitis (*n* = 6, 3.61%) and chemotherapy-related diarrhea (*n* = 6, 3.61%). Due to different levels of patient compliance, WMT treatment may not be completed on schedule. In this study, the time interval of WMT among enrolled patients was measured by the median number of days (25–75%); the baseline value was the laboratory result before the first course of treatment, and the median interval of 35 days (32–42, short term) after the second course of treatment, the median interval of 80 days (68.75–99.25, medium term) after the third course of treatment and the median interval of 188 days from baseline for the fourth course of treatment (154.75–207.50, long term) were used.

The comparison of demographic and clinical characteristics of patients grouped by BMI at baseline is shown in Table 2. Due to different levels of compliance, not all patients had complete data, so the number of patients in each index was different in each group. There was no significant difference in age, drinking history and smoking history between the overweight group and the normal-weight group, indicating that the basic situation of the study population was not different, which reduced the confounding factors in this study. There were significant differences between the following indexes and the normal weight group: BMI (27.38 ± 3.54 vs. 21.17 ± 1.65 kg/m^2^, *p* < 0.001), LFA (272.45 ± 34.65 vs. 221.22 ± 31.29 dB/m, *p* < 0.001), TC (5.20 ± 1.19 vs. 4.64 ± 0.99 mmol/L, *p* = 0.003), TG (3.85 ± 4.72 vs. 1.07 ± 0.62 mmol/L, *p* = 0.018), HDL-c (1.20 ± 0.31 vs. 1.33 ± 0.33 mmol/L, *p* = 0.026), ApoB (1.02 ± 0.21 vs. 0.89 ± 0.22 g/L, *p* = 0.001), non-HDL-C (4.00 ± 1.23 vs. 3.32 ± 0.92 mmol/L, *p* = 0.001), FBG (5.27 ± 1.40 vs. 4.72 ± 1.01 mmol/L, *p* = 0.012), FI (12.84 ± 7.37 vs. 6.80 ± 3.33 μU/mL, *p* < 0.011), HOMA-IR (3.19 ± 2.21 vs. 1.45 ± 0.80, *p* < 0.001), SBP (126.87 ± 13.01 vs. 120.89 ± 12.93 mmHg, *p* = 0.006), etc. but no significant differences between other indexes such as LSM (7.81 ± 2.67 vs. 6.59 ± 2.74 kPa, *p* = 0.060), LDL-c (3.09 ± 1.03 vs. 2.84 ± 0.87 mmol/L, *p* = 0.145), LIP (146.31 ± 144.60 vs. 144.80 ± 158.40 mmol/L, *p* = 0.974), HbA1c (6.44 ± 0.97 vs. 5.76 ± 0.84%, *p* = 0.099), DBP (78.94 ± 9.49 vs. 76.13 ± 9.40 mmHg, *p* = 0.077), etc.

### 3.2. Comprehensive Clinical Efficacy Evaluation of WMT for Overweight Patients

All enrolled patients were divided into the overweight group and normal-weight group according to obesity evaluation criteria. Patients were regrouped according to changes in BMI after WMT treatment (Table 3). The comprehensive curative effect of superrecombinant patients changed significantly during the course of treatment. Short-term normal recovery was 15.4% (*p* = 0.010), medium-term normal recovery was 20.00% (*p* = 0.114), long-term normal recovery was 30.00% (*p* = 0.210). The results showed that WMT can reduce BMI in overweight patients in the short, medium and long term. However, there was a significant difference in the short term, while there was no statistical difference in the medium and long term, which may be caused by the change in living habits and other factors during the treatment for as short as two months to as long as half a year. So, the efficacy of WMT remains to be explored. In conclusion, our data suggested that WMT had a significant short-term overall effect in overweight patients.

### 3.3. Comparative Analysis of Each Index after WMT Treatment and Baseline

**Figure 2:**
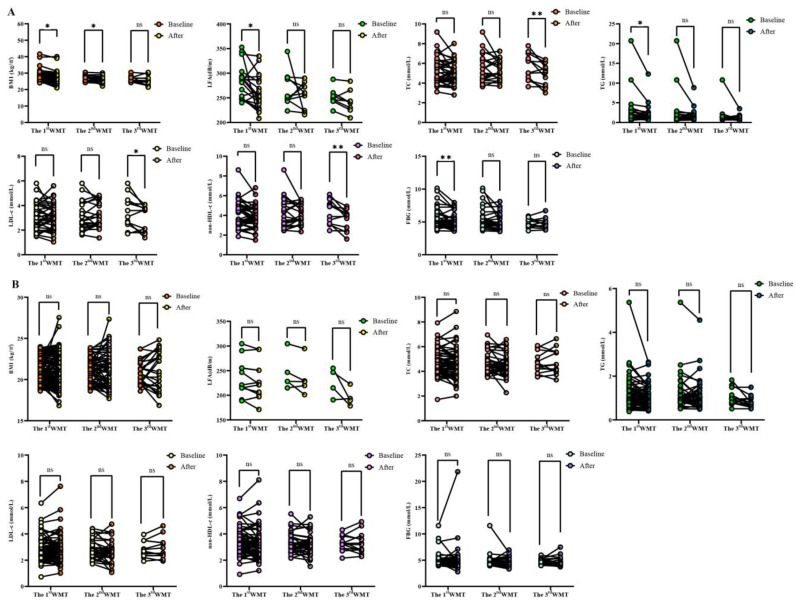
Changes in BMI, LFA, TC, TG, LDL-c, non-HDL-c and FBG levels after 1–3 WMTs. (**A**) Changes in BMI, LFA, TC, TG, LDL-c, non-HDL-c and FBG in OW group; (**B**) changes in BMI, LFA, TC, TG, LDL-c, non-HDL-c and FBG in NOW group. BMI, body mass index; LFA, liver fat attenuation; TC, total cholesterol; TG, triglyceride; LDL-c, low-density lipoprotein cholesterol; non-HDL-c, non-HDL cholesterol; FBG, fasting blood glucose. * indicates *p* < 0.05; ** indicates *p* < 0.01; ns, not significant.

Above, we observed that WMT had a significant improvement effect on overweight patients as a whole, and then we further analyzed specific indicators. Table 4 and Figure 2 show the effects of WMT on BMI, LFA, TC, TG, LDL-c, non-HDL-c, FBG, ALB and A/G in overweight patients. The results showed that after WMT, BMI decreased significantly in the short term (27.38 ± 3.54 to 26.73 ± 3.57 kg/m^2^, *p* = 0.004) and in the medium term (26.76 ± 1.71 to 25.78 ± 1.97 kg/m^2^, *p* = 0.012) (*p* < 0.05). In the long term (from 26.50 ± 1.93 to 25.50 ± 2.99 kg/m^2^, *p* = 0.253), there was also a reduction effect, but it was not significant due to the small number of people (*p* > 0.05). This suggested that WMT had a good effect on improving BMI in overweight patients. LFA decreased significantly in the short term (from 283.64 ± 34.72 to 262.14 ± 35.40 dB/m, *p* = 0.025) and, in the medium term (from 267.69 ± 35.60 to 254.99 ± 26.69 dB/m, *p* < 0.05). *p* = 0.402) and the long term (from 251.14 ± 18.16 to 242.20 ± 23.32 dB/m, *p* = 0.073), there was also a decreased effect, but because the number of people was too small, there was no significant effect in the medium and long term (*p* > 0.05). This indicated that WMT had a good effect on improving fatty liver in overweight patients. TC was significantly decreased in the long term (from 5.66 ± 1.35 to 4.87 ± 1.15 mmol/L, *p* = 0.007) (*p* < 0.05). TG significantly decreased in the short term (from 2.39 ± 3.51 to 1.81 ± 1.95 mmol/L, *p* = 0.036) (*p* < 0.05). LDL-c decreased significantly (from 3.49 ± 1.31 to 2.98 ± 1.05 mmol/L, *p* = 0.040) in the long term (*p* < 0.05). Non-HDL-c decreased significantly (from 4.32 ± 1.26 to 3.56 ± 1.04 mmol/L, *p* = 0.006) (*p* < 0.05). In general, WMT can significantly improve blood lipid in overweight patients. At the same time, WMT significantly decreased FBG in the short term (from 5.31 ± 1.46 to 4.91 ± 1.08 mmol/L, *p* = 0.005) during superrecombination (*p* < 0.05). It also showed a decreased effect in the medium term (5.62 ± 1.81 to 5.14 ± 1.24 mmol/L, *p* = 0.091) and in the long term (4.82 ± 0.70 to 4.75 ± 0.86 mmol/L, *p* = 0.726). However, there was no long-term significance due to the small number of people (*p* > 0.05). This indicated that WMT had a good effect on improving blood glucose in overweight patients. In the overweight group, WMT significantly decreased ALB in the long term (from 42.08 ± 3.27 to 39.61 ± 3.23 g/L, *p* = 0.012) (*p* < 0.05). A/G decreased significantly in the short term (from 1.52 ± 0.29 to 1.42 ± 0.21, *p* = 0.008) and the long term (from 1.74 ± 0.24 to 1.55 ± 0.31, *p* = 0.043) (*p* < 0.05). These results indicated that WMT can significantly improve liver function in overweight patients. In the normal-weight group, WMT caused no significant changes in BMI, LFA, TC, TG, LDL-c, HDL-c, non-HDL-c, FBG, ALB and A/G in the short, medium and long term, that is, WMT caused no significant changes in the normal-weight group.

### 3.4. Correlation Analysis of WMT on Overweight Index

**Figure 3:**
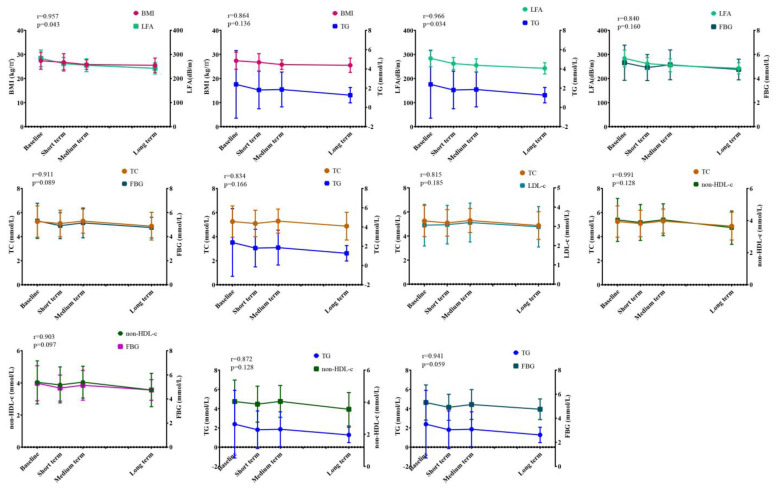
Correlation analysis of WMT on OW regulation. BMI, body mass index; LFA, liver fat attenuation; TC, total cholesterol; TG, triglyceride; LDL-c, low-density lipoprotein cholesterol; non-HDL-c, non-HDL cholesterol; FBG, fasting blood glucose. (r ≤ 0.3 indicates poor correlation; 0.3 < r ≤ 0.6 indicates moderately strong correlation; 0.6 < r ≤ 0.8 indicates strong correlation; r > 0.8 indicates extremely strong correlation).

Previously, we found that WMT significantly improved superrecombinant BMI, LFA, TC, TG, LDL-c, non-HDL-c, FBG, ALB and A/G during treatment. In order to find out the related factors affecting the regulation of WMT on overweight, correlation analysis was conducted on the above indicators with significant regulatory effects. As shown in Figure 3, we found that BMI had a strong positive correlation with LFA and TG in the overweight group. LFA was positively correlated with TG, FBG and ALB. TC was positively correlated with TG, LDL-c, non-HDL-c, FBG and ALB. There was a strong positive correlation between TG and non-HDL-c, FBG and ALB. Non-HDL-c showed strong positive correlation with FBG and ALB. FBG and ALB showed strong positive correlation. Our data showed that during the treatment of WMT, while improving the BMI of overweight patients, liver fat, blood lipid and blood glucose were also well improved, and BMI, liver fat, blood lipid and blood glucose were highly correlated. This provided us with a good therapeutic idea for the treatment of overweight or obesity, that is, WMT had a significant therapeutic effect on overweight patients. Besides weight loss, it can also play a comprehensive role in lowering blood glucose and lipid.

### 3.5. Prevalence of Adverse Events (AEs) in Patients Undergoing WMT

We also analyzed the prevalence of AEs in patients receiving WMT. A total of 460 WMT procedures were analyzed, and the overall incidence of AEs was 3.04%. Diarrhea was the most common AE (7, 1.52%), followed by abdominal pain (1, 0.22%), rash (1, 0.22%), dizziness (1, 0.22%), fatigue (1, 0.22%), nausea (1, 0.22%) and fever (1, 0.22%). In fact, these effects quickly resolved on their own after proper treatment or rest, and no serious adverse reactions were observed in any patients.

### 3.6. Analysis of Gut Microbiota Composition before and after WMT

**Figure 4:**
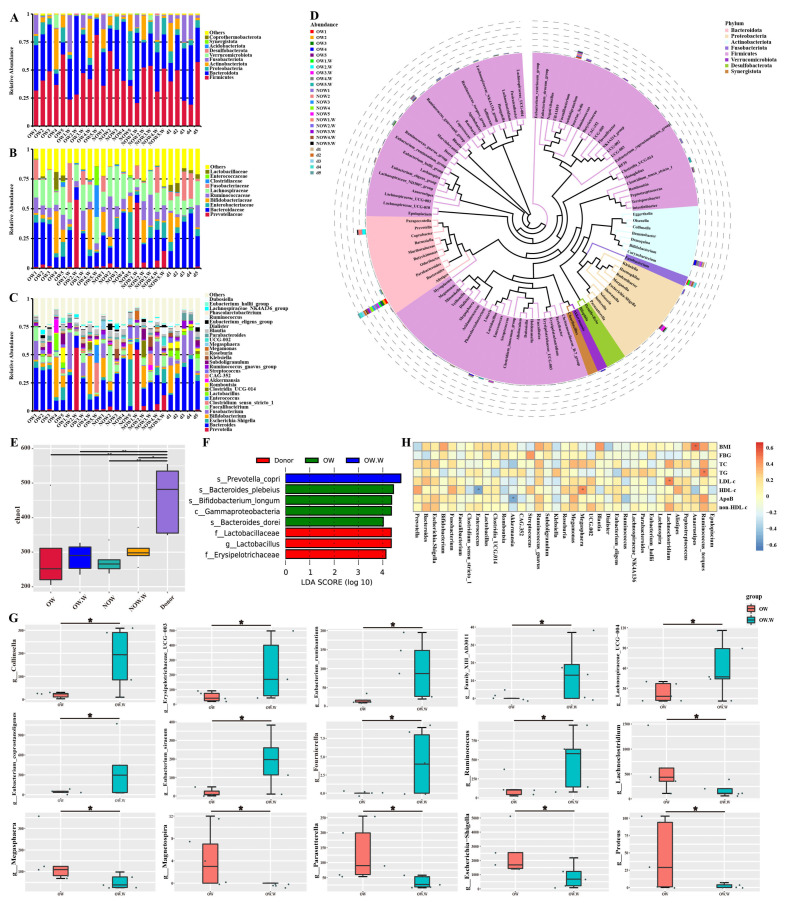
The composition of gut microbiota before and after WMT. (**A**) Composition of the top ten phyla of the gut microbiota. (**B**) The composition of gut microbiota in the top ten families. (**C**) The composition of gut microbiota in the top 30 genera. (**D**) Phylogenetic relationships of gut microbiota in the top 100 genera. (**E**) Chao1 index of α diversity analysis. (**F**) LEfSe analysis of OW group, OW.W group and donor group. (**G**) Metastat analysis before and after OW group WMT. (**H**) The interchanging relationship between environmental factors and species. OW: in OW group before WMT. OW.W: in OW group after WMT. NOW: in non-OW group before WMT. NOW.W: in non-OW group after WMT. BMI, body mass index; FBG, fasting blood glucose; TC, total cholesterol; TG, triglyceride; LDL-c, low-density lipoprotein cholesterol; HDL-c, high-density lipoprotein cholesterol; non-HDL-c, non-HDL cholesterol. * indicates *p* < 0.05; ** indicates *p* < 0.01.

We analyzed gut microbiota composition in the overweight (OW), normal-weight (NOW), and donor groups before and after WMT. At the phylum level, the gut microbiota mainly included *Firmicutes*, *Bacteroidota*, *Proteobacteria*, *Actinobacteriota*, *Fusobacteriota* and *Verrucomicrobiota*. At the phylum level, the relative abundance of *Firmicutes*, *Bacteroidota* and *Fusobacteriota* increased after WMT. The relative abundance of *Proteobacteria*, *Actinobacteriota* and *Verrucomicrobiota* was decreased (Figure 4A). At the family level, the relative abundance of *Prevotellaceae* and *Fusobacteriaceae* increased after WMT. The relative abundance of *Bacteroidaceae*, *Enterobacteriaceae* and *Lachnospiraceae* was reduced (Figure 4B). At the genus level, the relative abundance of *Prevotella*, *Fusobacterium* and *Enterococcus* was increased after WMT. The relative abundance of *Bacteroides*, *Escherichia*–*Shigella*, *Streptococcus* and *Klebsiella* was reduced (Figure 4C). For the normal-weight group, the relative abundance of *Prevotella*, *Lactobacillus* and *Akkermansia* beneficial bacteria increased after WMT at the genus level (Figure 4C). We analyzed phylogenetic relationships at the genus level for the top 100 gut microbiota. The top six were *Bacteroides, Prevotella*, *Escherichia-Shigella*, *Bifidobacterium*, *Fusobacterium* and *Faecalibacterium* (Figure 4D). Among them, WMT can increase their relative abundance, such as of *Prevotella*, *Lactobacillus* and *Akkermansia*, etc.

WMT increased gut microbiota α diversity in the overweight and normal-weight group, such as chao1 index (Figure 4E). LEfSe analysis was performed on the overweight group before and after WMT and the donor group to find the biomarkers with statistical differences between the groups. It was found that *Bacteroides plebeius*, *Bifidobacterium longum* and *Bacteroides dorei* were the distinct species before WMT in the overweight group, while *Prevotella copri* was the distinct species after WMT in the overweight group. The distinct species in the donor group was *Lactobacillus* (Figure 4F). The species with significant differences between the overweight group before and after WMT were identified by Metastat. We found that compared to baseline, WMT can significantly increase the relative abundance of *Collinsella*, *Erysipelotrichaceae* UCG−003, *Eubacterium ruminantium*, *Lachnospiraceae* UCG−004, *Eubacterium*, *Eubacterium coprostanoligenes*, *Eubacterium siraeum*, *Fournierella* and *Ruminococcus,* and significantly reduce the relative abundance of *Lachnoclostridium*, *Megasphaera*, *Magnetospira*, *Parasutterella*, *Escherichia*–*Shigella* and *Proteus* at the genus level in the overweight group gut microbiota (Figure 4G). Spearman rank correlation was used to study the mutual change relationship between environmental factors and species, and the correlation and significant *p* value between the two were obtained. We found a significant positive correlation between BMI and *Anaerostipes*. TG was positively correlated with *Ruminococcus* torques. LDL-c was positively correlated with *Lachnoclostridium*. HDL-c was significantly positively correlated with *Megasphaera* and negatively correlated with *Enterococcus*. ALB was positively correlated with *Lachnoclostridium* and *Bacteroides*. A/G showed a significant positive correlation with *Lachnospiraceae* NK4A136, *Roseburia* and *Bacteroides* and a significant negative correlation with *Bifidobacterium* (Figure 4H).

### 3.7. Analysis of Sphingolipid Metabolism before and after WMT

**Figure 5:**
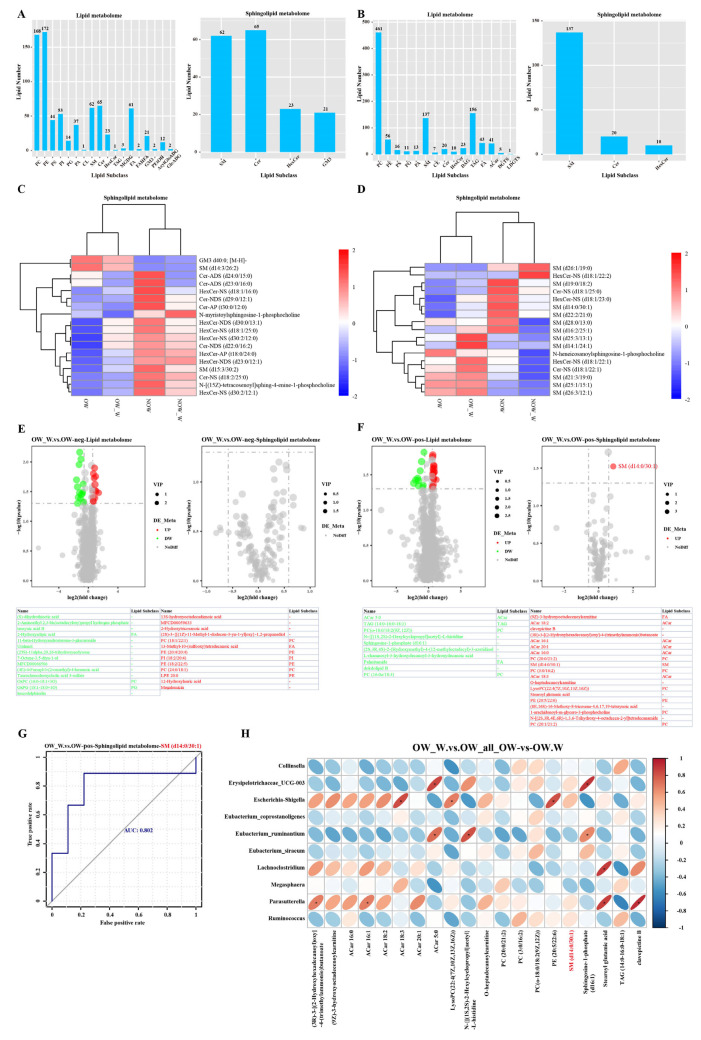
Analysis of lipid metabolomics and sphingolipid metabolomics in OW group. (**A**,**B**) Lipid metabolomics and sphingolipid metabolomics lipid subclass analysis. (**C**,**D**) Cluster heat map of different metabolites of sphingolipid metabolomics. (**C**) is anion mode, (**D**) is cationic mode. (**E**,**F**) Volcanic map analysis of lipid metabolomics and sphingolipid metabolomics. (**G**) Receiver operating characteristic analysis. (**H**) Correlation analysis of differential microflora and differential metabolites. The asterisk (*) indicates *p* < 0.05.

We studied the lipid metabolomics and sphingolipid metabolomics of serum in the overweight and normal-weight groups before and after WMT based on the LC-MS technique. In the anion mode of the OW group, lipid metabolomics mainly included 18 lipid subclasses including PC, PE, PS, etc. Sphingolipid metabolomics mainly consisted of four lipid subclasses: SM, Cer, HexCer and GM3 (Figure 5A). In the cationic mode, lipid metabolomics mainly consisted of 15 lipid subclasses, including PC, PE, PG, etc. Sphingolipid metabolomics mainly consisted of three lipid subclasses, SM, Cer and HexCer (Figure 5B). We conducted hierarchical cluster analysis on the differential metabolites obtained from the samples of the two groups, obtained the difference in metabolic expression patterns between and within the same comparison pair between the two groups and drew the differential metabolite cluster heat map. Sphingolipid metabolome analysis showed that in anion mode, differential metabolites included SM (d14:3/26:2), Cer-ADS (d24:0/15:0), HexCer-NDS (d30:0/13:1), etc. In the anion mode of the OW group, WMT can up-regulate metabolites such as HexCer-NDS (d30:0/13:1), HexCer-NS (d18:1/25:0) and Cer-NDS (d22:0/16:2) (Figure 5C). In the cationic mode, the differential metabolites included SM (d14:0/30:1), HexCer-NS (d18:1/22:2), Cer-NS (d18:1/25:0), etc. In the cationic mode of the OW group, WMT could up-regulate metabolites such as SM (d14:0/30:1), SM (d25:3/13:1), SM (d14:1/24:1), HexCer-NS (d18:1/22:1), Cer-NS (d18:1/22:1), etc. (Figure 5D).

The overall distribution of differential lipid compounds can be visually shown by volcanic maps. We found that in anion mode of the OW group, compared with baseline, WMT significantly up-regulated 13 metabolites, including PC (18:1/22:1), PE (20:4/20:4) and PI (18:2/20:4). It significantly down-regulated 14 metabolites including taurochenodeoxycholic acid 3-sulfate, 2-hydroxyadipic acid and OxPC (16:0–18:1 + 3O). Sphingolipid metabolome analysis showed no significant difference in sphingolipid metabolites (Figure 5E). In the cationic mode of the OW group, lipid metabolome analysis was compared with baseline. WMT can significantly up-regulate 13 metabolites including SM (d14:0/30:1), ACar 18:2, stearoyl glutamic acid and 1-arachidonoyl-sn-glycero-3-phosphocholine. Ten metabolites, such as ACar 5:0, sphingosine-1-phosphate (d16:1), palmitamide and L-rhamnosyl-3-hydroxydecanoyl-3-hydroxydecanoic acid, were significantly down-regulated. According to sphingolipid metabolome analysis, SM was only significantly up-regulated by WMT (d14:0/30:1) compared with baseline (Figure 5F). We further determined the accuracy of the ROC curve for SM (d14:0/30:1), a potential biomarker. Our results showed that the AUC value of SM (d14:0/30:1) was 0.802 (FIG. 5G), which had a certain prediction accuracy, indicating that our mined biomarkers were accurate. Finally, we conducted correlation analysis on the differential microflora and differential metabolites before and after WMT in the OW group and found that sphingomyelin (SM) (d14:0/30:1), *Escherichia–Shigella*, *Erysipelotrichaceae* UCG-003, *Ruminococcus*, *Parasutterella*, *Eubacterium siraeum*, *Collinsella* and *Eubacterium coprostanoligenes* were positively correlated. Sphingomyelin (SM) (d14:0/30:1) was negatively correlated with *Megasphaera*, *Eubacterium ruminantium* and *Lachnoclostridium*. Sphingosine-1-phosphate (d16:1), *Erysipelotrichaceae* UCG-003, *Eubacterium ruminantium*, *Eubacterium siraeum*, *Eubacterium coprostanoligenes*, *Ruminococcus* and *Collinsella* were positively correlated. Sphingosine-1-phosphate (d16:1) was negatively correlated with *Escherichia–Shigella*, *Megasphaera*, *Parasutterella* and *Lachnoclostridium* (Figure 5H).

## 4. Discussion

It was well known that the gut microbiome influences the host’s access to energy and energy storage from the diet [26]. Studies have shown that FMT from mice on a normal-fat diet into mice on a high-fat diet significantly reduces the body weight and metabolic characteristics of mice on a high-fat diet [27,28]. Obese subjects had a higher abundance of *Firmicutes* than *Bacteroidetes*, while leaner individuals had more *Bacteroidetes* and fewer *Firmicutes* [29,30]. Consistently, our results showed a similar effect in OW patients. The results showed that WMT can significantly improve BMI in OW patients in the short and medium term. This may be related to the increased abundance of beneficial gut microbiota after transplantation. It has been reported that the gut microbiota of the recipient after transplantation was similar to that of the donor [31]. Donor-specific microorganisms *Roseburia hominis, Ruminococcus lactaris* and *A. muciniphila* were able to successfully colonize the receptor, the latter being associated with improved host glucose tolerance [32]. These results suggested that FMT may be effective in the treatment of obesity by improving gut microbiota imbalance. Specifically, bacteria producing short-chain fatty acids (SCFAs) increased significantly after FMT, such as *Roseburia gutis, Bryantella forexigens* and *Megamonas hypermegale*, which may help improve insulin sensitivity in patients with abnormal glycolipid metabolism [33].

FMT can also improve plasma metabolic parameters in patients with abnormal glycolipid metabolism. In an equally interesting experiment, Sung et al. transplanted obese mice with fecal microbiomes from resveratrol-fed donor mice and normally fed donor mice. The results showed that compared with normally fed donor mice, the group of mice that received fecal microbial transplants from resveratrol-fed donors showed improvements in blood glucose levels [34]. Consistently, our results showed a similar effect in patients with overweight symptoms. These data lead us to conclude that transmission of beneficial bacteria or metabolites via FMT can improve blood glucose in patients with abnormal glucolipid metabolism.

Evidence has suggested that the gut microbiota plays an important role in the regulation of host energy metabolism and lipid levels [35,36]. Consistently, our results showed a similar effect in overweight patients. The results showed that WMT can significantly improve blood lipids in overweight patients. WMT can significantly reduce liver fat decay and triglycerides in overweight patients in the short term. It can significantly reduce total cholesterol, low-density lipoprotein and non-high-density lipoprotein in the long term. Therefore, it is promising to ameliorate these diseases and gut microbiota disorders by targeting the gut microbiota with probiotics or FMT.

Sphingolipids (SPLs) are involved in signal transduction inside and outside cells. Ceramide is associated with obesity [37]. Consistently, our results showed a similar effect in overweight patients. However, our study showed that WMT was the only significant up-regulator of sphingolipid (SM) (d14:0/30:1) compared with baseline by sphingolipid metabolome analysis. WMT can improve overweight clinically through sphingolipid metabolism, but further studies are needed to explore how WMT plays a role in weight loss and lipid reduction through sphingolipid (SM) (d14:0/30:1). In summary, the results seemed to suggest that further research is needed to explore the potential applications of SPL analysis to improve the prediction of risk associated with overweight and obesity in this population.

Overweight and obesity combine multiple symptoms. Therefore, a scientific and reasonable treatment strategy for overweight and obesity should be based on the control of blood glucose, blood lipids, blood pressure, weight and other measures. In our study, WMT significantly improved BMI, blood glucose and lipid levels in overweight patients. We hypothesize that the improvement in overweight after WMT is due to the improvement in gut microbiota after WMT. This is the same mechanism of improvement of WMT in patients with metabolic syndrome and hyperglycemia that we have shown before [38,39]. Overweight may be alleviated by synergies between intestinal symbiotic flora and sphingolipid metabolism after FMT treatment. At present, the understanding of the effect of WMT on metabolic diseases is still in its infancy, and the data on the effect of WMT on overweight are still lacking. This was a large-scale retrospective trial of overweight in south China, including both overweight and normal-weight groups. We established clinical evidence of the effects of WMT on overweight, which lays a foundation for subsequent studies on the effects of environmental factors [40], gut microbiota [41,42] and metabolic biomarkers [43] on abnormal glycolipid metabolism. Taken together, these results suggested that restoring gut microbiota can be a promising treatment for overweight; however, its mechanism needs further study.

The study had several limitations. Firstly, given that this was a retrospective study, more samples and data are needed to confirm the long-term efficacy of WMT in treating overweight. Secondly, the specific mechanism of action of WMT to improve overweight has not been elucidated. Third, we did not consider potential confounders between the primary symptoms of WMT treatment and overweight. In the future, we plan to conduct prospective studies with larger samples. Secondly, AI technology combined with metagenomics, transcriptomics, proteomics and metabolomics was used to analyze the comparative features before and after WMT to excavate the biomarkers of overweight and obesity and the specific gut microbiota and material basis of WMT. Finally, the mechanism of WMT on overweight was explored.

## 5. Conclusions

WMT had a significant improvement effect on OW patients. WMT can restore gut microbiota homeostasis in overweight patients and improve them by regulating sphingolipid metabolism. Therefore, the regulation of gut microbiota and sphingolipid metabolism by WMT may provide a new clinical approach for the treatment of overweight.

## Figures and Tables

**Table 1:** The main diagnoses of patients receiving washed microbiota transplantation.

Primary Cause for WMT	Number (*n*)	Percentage (%)
Functional bowel disease	85	51.20%
Ulcerative colitis	20	12.05%
Gastroesophageal reflux disease	17	10.24%
Non-alcoholic fatty liver disease	8	4.82%
Atopic dermatitis	6	3.61%
Chemotherapy-associated diarrhea	6	3.61%
Gouty arthritis	5	3.01%
Posthepatitic cirrhosis	3	1.81%
Crohn’s Disease	3	1.81%
Radiation enteritis	3	1.81%
Psoriasis vulgaris	1	0.60%
Hyperuricemia	1	0.60%
Depression	1	0.60%
Senile tremor	1	0.60%
Chronic urticaria	1	0.60%
Functional dyspepsia	1	0.60%
Bipolar disorder	1	0.60%
Perianal eczema	1	0.60%
Pustular psoriasis	1	0.60%
Neuromyelitis optica	1	0.60%
Total	166	100.00%

**Table 2:** Demographic and clinical characteristics of the patients at baseline according to BMI.

	Overweight Group (*n* = 52)	Normal-Weight Group (*n* = 114)	*p* Value
Age (year)	54.77 ± 16.74 (*n* = 52)	50.61 ± 15.31 (*n* = 114)	0.117
Drinking history *n* (%)	5 (9.62)	4 (3.51)	0.214
History of smoking *n* (%)	12 (23.08)	14 (12.28)	0.076
Male *n* (%)	34 (65.38)	49 (42.98)	0.007
BMI (kg/m^2^)	27.38 ± 3.54 (*n* = 52)	21.17 ± 1.65 (*n* = 114)	<0.001
LFA (dB/m)	272.45 ± 34.65 (*n* = 29)	221.22 ± 31.29 (*n* = 48)	<0.001
LSM (kPa)	7.81 ± 2.67 (*n* = 29)	6.59 ± 2.74 (*n* = 48)	0.060
TC (mmol/L)	5.20 ± 1.19 (*n* = 49)	4.64 ± 0.99 (*n* = 102)	0.003
TG (mmol/L)	2.18 ± 3.15 (*n* = 49)	1.07 ± 0.62 (*n* = 102)	0.018
LDL-c (mmol/L)	3.09 ± 1.03 (*n* = 49)	2.84 ± 0.87 (*n* = 102)	0.145
HDL-c (mmol/L)	1.20 ± 0.31 (*n* = 49)	1.33 ± 0.33 (*n* = 102)	0.026
ApoB (g/L)	1.02 ± 0.21 (*n* = 49)	0.89 ± 0.22 (*n* = 102)	0.001
non-HDL-c (mmol/L)	4.00 ± 1.23 (*n* = 49)	3.32 ± 0.92 (*n* = 102)	0.001
LIP (mmol/L)	146.31 ± 144.60 (*n* = 17)	144.80 ± 158.40 (*n* = 31)	0.974
FBG (mmol/L)	5.27 ± 1.40 (*n* = 52)	4.72 ± 1.01 (*n* = 112)	0.012
HbA1c (%)	6.44 ± 0.97 (*n* = 14)	5.76 ± 0.84 (*n* = 9)	0.099
FI (μU/mL)	12.84 ± 7.37 (*n* = 32)	6.80 ± 3.33 (*n* = 64)	<0.001
HOMA-IR	3.19 ± 2.21 (*n* = 32)	1.45 ± 0.80 (*n* = 62)	<0.001
SBP (mmHg)	126.87 ± 13.01 (*n* = 52)	120.89 ± 12.93 (*n* = 114)	0.006
DBP (mmHg)	78.94 ± 9.49 (*n* = 52)	76.13 ± 9.40 (*n* = 114)	0.077

Data presented as mean ± standard deviation or *n* (%). BMI (kg/m^2^), body mass index; LFA (db/m), liver fat attenuation; LSM (kPa), liver stiffness measurement; TC (mmol/L), total cholesterol; TG (mmol/L), triglyceride; LDL-c (mmol/L), low-density lipoprotein cholesterol; HDL-c (mmol/L), high-density lipoprotein cholesterol; ApoB (g/L), apolipoprotein B; non-HDL-c (mmol/L), non-HDL cholesterol; LIP (mmol/L), lipoprotein; FBG (mmol/L), fasting blood glucose; HbA1c (%), glycated hemoglobin; FI (μU/mL), fasting insulin; HOMA-IR, homeostasis model assessment of insulin resistance; SBP (mmHg), systolic blood pressure; DBP (mmHg), diastolic blood pressure.

**Table 3:** Comprehensive clinical efficacy of short-, medium- and long-term treatment on overweight.

Data Periods	Before Therapy (*n*)	Therapeutic Effect Base on Overweight			
		Unchanged Group (*n*)	Changed Group (*n*, %)	X^2^	*p* Value
OW Group					
Short term	52	44	8 (15.4%)	6.635	0.010
Medium term	20	16	4 (20.0%)	2.500	0.114
Long term	10	7	3 (30.0%)	1.569	0.210

The definitions of unchanged and changed of OW group were still in OW group and changed to NOW group.

**Table 4:** The comparison values of each index in OW group and NOW group in the short term, medium term and long term with baseline during the treatment of washed microbiota transplantation.

Items	Baseline	Short Term	*p* Value	Baseline	Medium Term	*p* Value	Baseline	Long Term	*p* Value
OW Group									
BMI (kg/m^2^)	27.38 ± 3.54 (*n* = 52)	26.73 ± 3.57 (*n* = 52)	0.004	26.76 ± 1.71 (*n* = 20)	25.78 ± 1.97 (*n* = 20)	0.012	26.50 ± 1.93 (*n* = 10)	25.50 ± 2.99 (*n* = 10)	0.253
LFA (dB/m)	283.64 ± 34.72 (*n* = 18)	262.14 ± 35.40 (*n* = 18)	0.025	267.69 ± 35.60 (*n* = 10)	254.99 ± 26.69 (*n* = 10)	0.402	251.14 ± 18.16 (*n* = 8)	242.20 ± 23.32 (*n* = 8)	0.073
LSM (kPa)	8.21 ± 2.76 (*n* = 18)	7.22 ± 2.96 (*n* = 18)	0.277	7.26 ± 2.16 (*n* = 10)	6.93 ± 1.75 (*n* = 10)	0.747	7.69 ± 2.38 (*n* = 8)	8.03 ± 2.35 (*n* = 8)	0.728
TC (mmol/L)	5.25 ± 1.30 (*n* = 38)	5.09 ± 1.10 (*n* = 38)	0.279	5.47 ± 1.46 (*n* = 21)	5.29 ± 1.00 (*n* = 21)	0.402	5.66 ± 1.35 (*n* = 12)	4.87 ± 1.15 (*n* = 12)	0.007
TG (mmol/L)	2.39 ± 3.51 (*n* = 38)	1.81 ± 1.95 (*n* = 38)	0.036	2.66 ± 4.67 (*n* = 21)	1.86 ± 1.81 (*n* = 21)	0.235	2.01 ± 2.79 (*n* = 12)	1.27 ± 0.80 (*n* = 12)	0.254
LDL-c (mmol/L)	3.06 ± 1.08 (*n* = 38)	3.09 ± 1.00 (*n* = 38)	0.843	3.14 ± 1.18 (*n* = 21)	3.20 ± 1.01 (*n* = 21)	0.666	3.49 ± 1.31 (*n* = 12)	2.98 ± 1.05 (*n* = 12)	0.040
HDL-c (mmol/L)	1.2 ± 0.32 (*n* = 38)	1.21 ± 0.3 (*n* = 38)	0.749	1.28 ± 0.36 (*n* = 21)	1.23 ± 0.30 (*n* = 21)	0.227	1.34 ± 0.27 (*n* = 12)	1.32 ± 0.28 (*n* = 12)	0.562
ApoB (g/L)	1.04 ± 0.21 (*n* = 38)	1.04 ± 0.24 (*n* = 38)	0.832	1.02 ± 0.19 (*n* = 21)	1.09 ± 0.20 (*n* = 21)	0.044	1.09 ± 0.21 (*n* = 12)	1.05 ± 0.20 (*n* = 12)	0.375
non-HDL-c (mmol/L)	4.04 ± 1.34 (*n* = 38)	3.88 ± 1.12 (*n* = 38)	0.229	4.19 ± 1.57 (*n* = 21)	4.05 ± 0.99 (*n* = 21)	0.543	4.32 ± 1.26 (*n* = 12)	3.56 ± 1.04 (*n* = 12)	0.006
LIP (mmol/L)	147.96 ± 151.03 (*n* = 14)	166.56 ± 168.51 (*n* = 14)	0.034	258.35 ± 71.77 (*n* = 2)	267.35 ± 23.26 (*n* = 2)	0.837	/	/	/
FBG (mmol/L)	5.31 ± 1.46 (*n* = 46)	4.91 ± 1.08 (*n* = 46)	0.005	5.62 ± 1.81 (*n* = 24)	5.14 ± 1.24 (*n* = 24)	0.091	4.82 ± 0.70 (*n* = 11)	4.75 ± 0.86 (*n* = 11)	0.726
HbA1c (%)	6.53 ± 0.57 (*n* = 3)	6.63 ± 0.81 (*n* = 3)	0.622	7.75 ± 0.50 (*n* = 2)	7.70 ± 0.28 (*n* = 2)	0.942	/	/	/
FI (μU/mL)	12.27 ± 6.83 (*n* = 22)	12.57 ± 5.16 (*n* = 22)	0.835	10.38 ± 5.11 (*n* = 12)	10.15 ± 5.01 (*n* = 12)	0.809	11.82 ± 5.43 (*n* = 8)	12.45 ± 5.44 (*n* = 8)	0.628
HOMA-IR	3.02 ± 2.24 (*n* = 22)	2.89 ± 1.61 (*n* = 22)	0.813	2.47 ± 1.40 (*n* = 12)	2.32 ± 1.19 (*n* = 12)	0.533	2.55 ± 1.47 (*n* = 7)	2.59 ± 1.35 (*n* = 7)	0.902
SBP (mmHg)	126.87 ± 13.01 (*n* = 52)	125.06 ± 10.65 (*n* = 52)	0.321	125.48 ± 13.82 (*n* = 25)	123.12 ± 11.66 (*n* = 25)	0.451	121.38 ± 10.09 (*n* = 13)	119.69 ± 10.70 (*n* = 13)	0.713
DBP (mmHg)	78.94 ± 9.49 (*n* = 52)	78.4 ± 8.86 (*n* = 52)	0.759	78.64 ± 10.46 (*n* = 25)	76.12 ± 8.21 (*n* = 25)	0.252	75.85 ± 9.60 (*n* = 13)	80.08 ± 7.78 (*n* = 13)	0.223
**NOW Group**									
BMI (kg/m^2^)	21.15 ± 1.65 (*n* = 113)	21.22 ± 2.00 (*n* = 113)	0.609	21.22 ± 1.56 (*n* = 64)	21.16 ± 2.13 (*n* = 64)	0.760	20.79 ± 1.42 (*n* = 24)	21.07 ± 2.35 (*n* = 24)	0.399
LFA (dB/m)	236.34 ± 37.79 (*n* = 11)	230.38 ± 38.60 (*n* = 11)	0.101	244.56 ± 35.42 (*n* = 5)	234.43 ± 35.47 (*n* = 5)	0.149	226.85 ± 29.56 (*n* = 4)	195.93 ± 18.87 (*n* = 4)	0.117
LSM (kPa)	7.58 ± 3.97 (*n* = 11)	6.67 ± 2.39 (*n* = 11)	0.437	7.94 ± 5.22 (*n* = 5)	6.89 ± 3.20 (*n* = 5)	0.560	9.43 ± 5.09 (*n* = 4)	8.57 ± 6.53 (*n* = 4)	0.714
TC (mmol/L)	4.65 ± 1.01 (*n* = 70)	4.58 ± 1.12 (*n* = 70)	0.350	4.63 ± 0.84 (*n* = 38)	4.49 ± 0.88 (*n* = 38)	0.250	4.69 ± 0.83 (*n* = 13)	4.75 ± 1.02 (*n* = 13)	0.688
TG (mmol/L)	1.15 ± 0.70 (*n* = 70)	1.03 ± 0.47 (*n* = 70)	0.053	1.21 ± 0.83 (*n* = 38)	1.14 ± 0.76 (*n* = 38)	0.288	1.04 ± 0.38 (*n* = 13)	0.84 ± 0.27 (*n* = 13)	0.059
LDL-c (mmol/L)	2.84 ± 0.90 (*n* = 70)	2.80 ± 1.04 (*n* = 70)	0.601	2.78 ± 0.70 (*n* = 38)	2.67 ± 0.80 (*n* = 38)	0.287	2.83 ± 0.60 (*n* = 13)	2.90 ± 0.80 (*n* = 13)	0.434
HDL-c (mmol/L)	1.30 ± 0.33 (*n* = 70)	1.31 ± 0.35 (*n* = 70)	0.808	1.32 ± 0.37 (*n* = 38)	1.31 ± 0.27 (*n* = 38)	0.742	1.39 ± 0.38 (*n* = 13)	1.45 ± 0.34 (*n* = 13)	0.106
ApoB (g/L)	0.89 ± 0.24 (*n* = 70)	0.88 ± 0.26 (*n* = 70)	0.665	0.90 ± 0.18 (*n* = 38)	0.88 ± 0.20 (*n* = 38)	0.481	0.88 ± 0.16 (*n* = 13)	0.90 ± 0.23 (*n* = 13)	0.565
non-HDL-c (mmol/L)	3.35 ± 0.95 (*n* = 70)	3.27 ± 1.10 (*n* = 70)	0.292	3.31 ± 0.72 (*n* = 38)	3.18 ± 0.77 (*n* = 38)	0.220	3.30 ± 0.58 (*n* = 13)	3.30 ± 0.80 (*n* = 13)	0.972
LIP (mmol/L)	68.94 ± 62.60 (*n* = 11)	64.62 ± 75.96 (*n* = 11)	0.696	75.15 ± 67 (*n* = 8)	60.49 ± 48.7 (*n* = 8)	0.351	109.25 ± 44.48 (*n* = 2)	90.90 ± 70.43 (*n* = 2)	0.500
FBG (mmol/L)	4.72 ± 1.03 (*n* = 97)	4.70 ± 1.93 (*n* = 97)	0.886	4.71 ± 1.06 (*n* = 53)	4.53 ± 0.77 (*n* = 53)	0.172	4.61 ± 0.54 (*n* = 23)	4.70 ± 0.84 (*n* = 23)	0.533
HbA1c (%)	5.15 ± 0.50 (*n* = 2)	5.20 ± 0.71 (*n* = 2)	0.795	/	/	/	/	/	/
FI (μU/mL)	6.93 ± 3.84 (*n* = 38)	6.91 ± 3.44 (*n* = 38)	0.970	7.52 ± 3.82 (*n* = 14)	9.37 ± 5.82 (*n* = 14)	0.208	8.45 ± 5.08 (*n* = 6)	6.49 ± 2.18 (*n* = 6)	0.319
HOMA-IR	1.55 ± 0.95 (*n* = 37)	1.60 ± 1.28 (*n* = 37)	0.755	1.76 ± 1.07 (*n* = 14)	2.16 ± 1.71 (*n* = 14)	0.171	1.77 ± 1.22 (*n* = 6)	1.33 ± 0.55 (*n* = 6)	0.301
SBP (mmHg)	120.89 ± 12.93 (*n* = 114)	118.92 ± 10.83 (*n* = 114)	0.133	120.51 ± 13.75 (*n* = 65)	120.18 ± 12.09 (*n* = 65)	0.866	119.96 ± 10.72 (*n* = 24)	119.58 ± 9.32 (*n* = 24)	0.858
DBP (mmHg)	76.13 ± 9.4 (*n* = 114)	75.7 ± 8.64 (*n* = 114)	0.649	75.57 ± 9.35 (*n* = 65)	75.11 ± 8.00 (*n* = 65)	0.734	75.42 ± 7.18 (*n* = 24)	75.67 ± 8.47 (*n* = 24)	0.878

Data presented as mean ± standard deviation or *n* (%).

## Data Availability

The original contributions presented in the study are publicly available. These data can be found in the NCBI Sequence Read Archive (accession: PRJNA916000).
